# Sleep-Related Offline Learning in a Complex Arm Movement Sequence

**DOI:** 10.2478/hukin-2014-0002

**Published:** 2014-04-09

**Authors:** Andreas Malangré, Peter Leinen, Klaus Blischke

**Affiliations:** 1 Institute of Sport Science, Saarland University, Saarbruecken, Germany.

**Keywords:** motor learning, sleep, memory consolidation, motor sequence, gross motor skill

## Abstract

Sleep is known to elicit off-line improvements of newly learned procedural skills, a phenomenon attributed to enhancement consolidation of an internal skill representation. In the motor domain, enhancement consolidation has been reported almost exclusively for sequential-finger-tapping skills. The aim of the present study was to extend the notion of sleep-related enhancement consolidation to tasks closer to everyday motor skills. This was achieved by employing a sequence of unrestrained reaching-movements with the non-dominant arm. Fifteen reaching-movements had to be executed as fast as possible, following a spatial pattern in the horizontal plane. Terminating each movement, a peg had to be fitted into a hole on an electronic pegboard. Two experimental groups received initial training, one in the evening, the other one in the morning. Subsequently, performance in both groups was retested twelve, and again 24 hrs later. Thus, during retention each individual experienced a night of sleep, either followed or preceded by a wake interval. Performance error remained low throughout training and retests. Yet mean total execution time, indicative of task execution-speed, significantly decreased for all individuals throughout initial training (no group differences), and significantly decreased again in either group following nocturnal sleep, but not following wake. This finding does not appear to result merely from additional practice afforded at the time of retests, because only after a night of sleep individuals of both experimental groups also revealed performance improvement beyond that estimated from their initial training performance.

## Introduction

Today, there is a considerable body of research in the neurobehavioral sciences addressing the consolidation and optimization of internal representations in the course of motor learning (Song, 2009). In particular it has been shown that after initial practice of a motor skill (and in the absence of any further physical practice) the elapse of time stabilizes performance, while sleep or daytime naps administered during the retention interval result in an additional performance enhancing effect. During the last decade this phenomenon, usually referred to as “offline learning” or “enhancement consolidation” (EC), has been corroborated by numerous behavioral studies (Fischer et al., 2005; Walker, 2005).

However, nearly all of these studies employed very similar types of tasks frequently used in the domain of motor sequence learning: namely the serial-reaction-time task (SRTT) or the sequential-finger-tapping task (Albouy et al., 2013; Friedman and Korman, 2012). And although it was proclaimed ten years ago already that “this finding of sleep-dependent motor skill improvement may have important implications for the efficient learning of *all* skilled actions in humans” (Walker et al., 2002), even today there is still little evidence to support that claim. This is the more surprising, since practitioners in the applied field of movement studies (i.e. coaches and therapists) would readily introduce the notion of sleep-related offline learning to their practice routines, was it only confirmed with respect to tasks immediately relevant to their vocational domains. So the question still needs to be answered, as to what extent the above findings also apply to motor tasks beyond sequential-finger-tapping skills.

In the first attempt to systematically address this issue, we conducted a series of experiments involving different motor criterion tasks (Blischke et al., 2008). In these experiments, sleep-related offline learning in the standard sequential-finger-tapping task was successfully replicated. By contrast, sleep-related EC was neither observed (a) in a relative timing task incorporating an integer rhythm, nor (b) in a pursuit-tracking task with subjects being *un*aware of the spatial pattern to be learned, nor (c) when subjects had to precisely (re)produce a sub-maximal force impulse in a counter movement jump. While EC in rhythmic movements still is a matter of debate (Lewis et al., 2011; Wright et al., 2012), it has been argued elsewhere that sleep-related EC essentially depends on explicit (sequence) knowledge and awareness (Robertson et al., 2004). This plus assuming force impulses being represented in a rather implicit fashion, may well account for the absence of offline learning in tasks like pursuit-tracking and ballistic force production.

So at first perusal it seems that sleep-related EC requires some involvement of *declarative* memory processes, often associated with routines of *explicit* learning. Moreover, as perceptual skill learning (like in visuo-motor adaptation tasks) has been found to be rather sleep-independent (Doyon et al., 2009; but see Huber et al., 2004), sleep-related EC should be most pronounced in movement *sequences*, organized in *allocentric space* (Albouy et al., 2013; Cohen et al., 2005; Witt et al., 2010). Namely, fast and precise sequence execution requires rapid *in-advance specification* of requisite elements to be organized in immediate succession (Rhodes et al., 2004; Verwey, 1996). This process would be facilitated if spatial memory reorganization occurred across a period of sleep (Kuriyama et al., 2004). If all this holds true, then also *gross* motor tasks relevant to sports, occupational therapy, and motor rehabilitation should be amenable to sleep-related EC, if they were only *spatially* defined, *sequentially* organized, and *explicitly* acquired.

This notion was initially tested in a study by Schmidt et al. (2010). Following a SRTT-paradigm, subjects were required to repeatedly produce a sequence of unrestrained arm movements: on a vertically positioned smart board, four horizontally aligned rectangles had to be touched with the hand(s) as fast as possible, thereby following a certain order. Actually, this task was an enlarged version of the same spatial pattern incorporated in finger-tapping tasks, which repeatedly had been proven subject to sleep-related EC when being executed on a key-board (Walker, 2005; Blischke et al., 2008). Two experiments were run on the smart board, with participants using either one or both hands. However, in contrast to the key-pressing studies, *no* sleep-related offline learning was found in any of these experiments incorporating gross limb movements instead of just activating different digits in a certain order (Schmidt et al., 2010).

At this point one might conclude that findings on sleep-related EC in sequential finger-tapping skills in fact do not generalize to gross motor tasks at all, suggesting some principal dissociation of fine and gross motor sequence representation. However, considering the impact motor skill complexity may have on sleep-dependent learning (Kuriyama et al., 2004), perhaps the arm-movement sequence in Schmidt and colleagues’ study was just lacking the necessary amount of task difficulty: first, task complexity was low in general as the sequence incorporated just five elements. Also, precision requirements were insignificant considering the generous dimensions of the spatial goals on the smart board. With cognitive requirements and motor control demands being that low, any actual memory enhancement therefore might not have come overtly into effect at sequence recall tests. Moreover, as was suggested by individual reports, performance improvements due to EC could have been masked by muscular fatigue as subjects had to continuously produce rapid full-range arm movements in the frontal plane for 30 seconds at a time. Thus with increasing skill expertise and execution speed, subjects’ performance might have approached a physically determined ceiling effect.

As a consequence, another attempt at investigating sleep-related EC in gross-motor tasks was undertaken by introducing modified task conditions. In the study presented here, an unrestrained arm-movement sequence to be executed as fast as possible was employed again. However, to increase task difficulty the sequence this time comprised a series of 15 reaching movements (i.e. sequence elements) with the (non-dominant) hand serving as a single end-effector. Also, drawing on the systematic effect of target width and movement amplitude on movement time first mathematically analyzed by Fitts (1954), precision requirements for all sequence elements were set at an index of difficulty (ID) of 4.95 on average, with IDs > 4.5 being regarded as high (Boyle and Shea, 2011). According to Fitts, the ID is determined by the equation Log_2_(2A/W), where A represents the movement amplitude measured from one target center to the other target center and W represents the width of the target area in the direction of the movement. Furthermore, in order to somewhat reduce muscular fatigue the movements this time had to be carried out in the horizontal plane. Also, unlike in the SRTT-paradigm each sequence execution trial now was triggered by a separate start signal. So the present task to some extent resembled features of a discrete sequence production task (DSPT; Rhodes et al., 2004), thereby reducing once more the chance of fatigue building up within blocks of successive trials. Altogether the gross-motor task employed this time appears to be largely cleared of those features which possibly diminished effects of sleep-related EC in earlier studies, yet at the same time still bears good resemblance to sport skills and activities of daily living.

Thus, with respect to this gross-motor task comprised by a sequence of unrestrained arm-movements, it was hypothesized that after initial learning *sleep*, but not wake, significantly facilitates performance (namely: execution speed) at retention beyond any improvement merely being afforded by exposure to additional practice trials.

## Material and Methods

### Subjects

24 subjects (25.5 ± 3.9 years; 6 females, 1 left-handed; 18 males) participated in this study, which was conducted in accordance with the ethical standards of the 1964 Declaration of Helsinki. All participants gave their written informed consent. Subjects were randomly assigned to two experimental groups, the EME (= *E*vening-*M*orning-*E*vening)-group (N = 12) and the MEM (= *M*orning-*E*vening-*M*orning)-group (N = 12). Sample sizes were chosen so that possible performance enhancements at retention should turn out significant if they were at least the size of those encountered previously for the standard finger-tapping task, which yielded η^2^_p_-values of ≥ .326 in the MEM-groups and ≥ .519 in the EME-groups for improvements in execution speed (Blischke et al., 2008). Since previous studies showed that performance is unaffected by gender, experimental groups were not balanced with respect to sex of participants. Participants were required to refrain from daytime naps, alcohol, excessive caffeine-intake, and any other drugs from the night before training session until the end of the experiment. Physical activity (e.g. sport practice) was permitted.

### Task and dependent measures

Holding a small peg with their non-dominant hand, on a start signal subjects were to carry out a sequence of 15 arm movements in the horizontal plane. Following a fixed pattern of end-point locations, these movements differed in range (from 3.83 to 33.75 cm) as well as in direction (Picture 1, lower panel). With their hand visible all the time, at the end of each movement participants had to quickly fit the peg into a hole of a pegboard in front of them, thereby closing a magnetic contact. The pegboard employed consisted of two horizontal wooden bars (41.7 cm long, 16 cm apart), each containing ten holes 22.22 mm in depth, 12.7 mm in diameter, and 25.4 mm apart in the left-right dimension, 195 mm apart in the forward-backward dimension (Picture 1). On a computer screen representing the 20 pegboard holes, each present movement goal was illuminated red until the respective magnetic contact was closed, turning the color from red to green. At the same time the next goal was indicated by turning to red. Once the respective sequence element was terminated, the next movement had to be started immediately, until the sequence was completed. No additional information (e.g. augmented feedback) was provided. As participants could use their whole arm by freely moving shoulder, elbow and wrist, they were required to control redundant biomechanical degrees of freedom. Subjects were instructed to carry out each single sequence-trial as fast and with as few errors as possible, and not to speed up performance at the risk of increasing number of errors. Dependent measures taken for each subject were *Total Execution Time* (TET) per sequence, averaged over the number of correct sequences per trial block, and the number of *Erroneous Sequences* (ES) per trial block. Thus, TET is inversely proportional to sequence execution speed.

### Design and procedure

After being shortly familiarized with the electronical pegboard and the peg-plugging procedure in general, both experimental groups received initial training of the criterion task (ten blocks of ten trials each), the EME-group in the evening (7 to 9 p.m.), the MEM-group in the morning (7 to 9 a.m.). Both groups then were retested 12 hrs (Retest 1), and once again 24 hrs later (Retest 2), with each Retest comprising three blocks of ten trials. Thus, subjects in the EME-group had a regular night’s sleep during their first, those in the MEM-group during their second retention interval. Trial blocks always were separated by a pause of 30 seconds during practice as well as in Retests.

### Statistics

First, for each subject ES- and TET-measures were averaged across trials per block. Then for each subject and dependent variable, a “Post-Training” measure was calculated from the last three initial training blocks (blocks 8, 9 & 10), while Retest 1- and Retest 2-measures were calculated from blocks 11, 12 & 13, and 14, 15 & 16, respectively. Group means were calculated on this basis. For inferential statistics, two-way ANOVAs on the factors “Group” x “Acquisition-Block” and “Group” x “Test” (levels: Post-Training, Retest 1, Retest 2), one-way ANOVAs on the factor “Test”, and paired t-tests were run. With respect to repeated-measures factors, in case of violation of the sphericity assumption *df*-correction according to Greenhouse-Geisser was applied. A significance level of *p* ≤ .05 was used for all inferential statistics. Calculations were conducted with SPSS-PC, version 15.0. Effect sizes were provided in terms of η^2^_p_ with respect to ANOVAs, and Cohen’s *d* with respect to t-tests.

## Results

### Performance during acquisition

**Figure 1.**
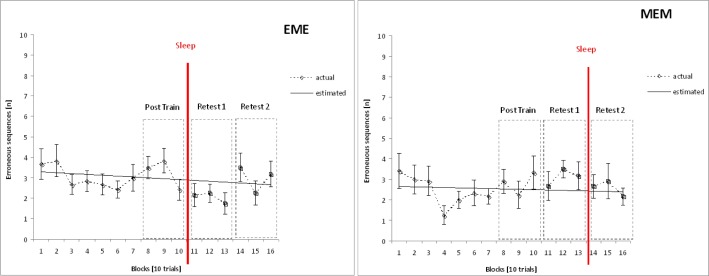
Number of Erroneous Sequences (ES) per trial block: initial training (blocks 1 through 10) and subsequent retests. Symbols represent group means per trial block (actual performance); error bars: standard errors of the mean. Solid lines represent linear functions derived from group mean initial training data (EME: y = −0.042x + 3.31; MEM: y = −0.018x + 2.66). Left panel: EME-group; right panel: MEM-group.

**Figure 2.**
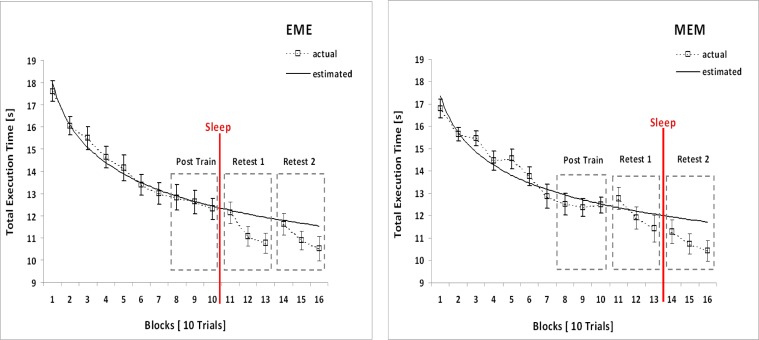
Mean Total Execution Time (TET; seconds) per trial block (correct sequences only): initial training (blocks 1 through 10) and subsequent retests. Symbols represent group means per trial block (actual performance); error bars: standard errors of the mean. Solid lines represent power functions derived from the group mean initial training data (EME: y = 17.97x ^−0.16^, R^2^ = 0.98; MEM: y = 17.36x ^−0.142^, R^2^ = 0.92). Left panel: EME-group; right panel: MEM-group.

To assess changes in performance during initial training, 2[Group] × 10[Block]-ANOVAs (repeated-measures factor “Block”) were calculated on the respective data (Figures 1 and 2). On the average, there are 2.8 (± 2.01) *Erroneous Sequences* (ES) per trial block (EME: 3.08 ± 2.1; MEM: 2.56 ± 2.16). Error rate is small from the beginning and does not fluctuate significantly across acquisition (*p* = .079, η^2^_p_ = .085). There is no Group x Block interaction (*p* = .273, η^2^_p_ = .054), nor do groups differ in performance (*p* = .374, η^2^_p_ = .036). In contrast, *Total Execution Time* (TET) significantly decreases during acquisition in both experimental groups (*F*_[Block]_ (4.304, 94.690) = 98.053, *p* < .001, η^2^_p_ = .817). Again there is no Group x Block interaction (*p* = .295, η^2^_p_ = .054), nor do groups differ (*p* = .832, η^2^_p_ = .002).

### Comparing performance at the end of practice and retention

To asses possible changes in performance across the two retention intervals, 2[Group] × 3[Test] ANOVAs with “Test” as a repeated-measures factor (levels: Post-Training, Retest 1, Retest 2) were calculated on the respective data (Figures 1 and 2). In accord with our central hypothesis, significant Group × Test interactions (in combination with significant pairwise comparisons concerning the interaction term) were taken as evidence for sleep-related EC in a particular retention interval if, according to the descriptive data for those participants who had slept during that interval, improvements in performance were indeed present and at the same time larger than those for subjects who had stayed awake during the same period of time.

In such a case, however, in order to unambiguously attribute possible changes in performance to the exact succession of wake and sleep periods, data were also analyzed separately for each circadian condition (EME and MEM) by means of repeated-measures one-way-ANOVAs on the factor “Test”. Only this way it is possible to unequivocally dissociate within each group periods reflecting true performance improvements from periods with performance remaining more or less the same, thus making allowance for the fact that by theory (Walker, 2005) enhancement consolidation is attributed to sleep, but not just to the elapse of time.

#### Performance errors

According to a 2[Group] x 3[Test] ANOVA, the overall number of Erroneous Sequences (ES) does not change across tests at all (*p*_[Test]_ = .239, η^2^_p_ = .063), nor do groups differ on the whole (*p*_[Group]_ = .904, η^2^_p_ = .001). There is, however, a significant Group x Test interaction (*F* (1.575, 34.658) = 4.996, *p* = .018, η^2^_p_ = .185). Also, each of the respective pairwise comparisons turns out to be significant (Post-Training, Retest 1: *F* (1, 22) = 7.650, *p* = .011, η^2^_p_ = .258; Retest 1, Retest 2: *F* (1, 22) = 5.067, *p* = .035, η^2^_p_ = .187). However, considering the somewhat ambiguous descriptive data (Figure 1), even in the light of these results the following questions still remained to be answered: (a) is error reduction observed in the EME-group following sleep at Retest 1, which is indicative of sleep-related EC, completely lost again during following wake-interval? And, (b) does error rate in the MEM-group change at all? Thus for further clarification, repeated-measures one-way-ANOVAs on the factor “Test” were run for each of the experimental groups separately.

As is corroborated this way, the error rate significantly changes during retention in the EME-group only (*F*_[Test]_ (2, 22) = 4.948, *p* = .017, η^2^_p_ = .310). According to the respective pairwise comparisons (i.e. within-subjects contrasts), for this group a significant reduction in errors occurs during the *first* 12-hr retention interval, i.e. following *sleep* (Post-Training, Retest 1: *F* (1, 11) = 12.008, *p* = .005, η^2^_p_ = .522). During the following wake period the error rate increases somewhat again indeed, yet without this effect reaching statistical significance (Retest 1, Retest 2: *p* = .088, η^2^_p_ = .242). However, in the MEM-group, the respective ANOVA covering the total 24-hr retention interval does not yield significance altogether (*p* = .332. η^2^p = .091).

#### Execution time

According to a 2[Group] x 3[Test] ANOVA, Total Execution Time (TET) significantly decreases across tests in both groups (*F*_[Test]_ (2, 44) = 45.590, *p* < .001, η^2^_p_ = .675), while groups as such do not differ (*p*_[Group]_ = .843, η^2^_p_ = .002). Again there is a significant Group x Test interaction (*F* (2, 44) = 4.308, *p* = .020, η^2^_p_ = .164). According to the respective pairwise comparisons, this interaction pertains as well to the first (*F* (1, 22) = 6.071, *p* = .022, η^2^_p_ = .216) as to the second 12-hr retention interval (*F* (1, 22) = 11.442, *p* = .003, η^2^_p_ = .342). Considering the descriptive data (Figure 2), these results strongly support our central assumption of sleep-related EC coming into place in both experimental groups in terms of significantly shorter TET, that is increased execution speed. At this point it remains unclear, however, if TET in both groups also decreases significantly, though to a lesser extent, during their respective wake intervals. Thus, again, repeated-measures one-way-ANOVAs on the factor “Test” were run for each of the experimental groups separately.

**Figure 3.**
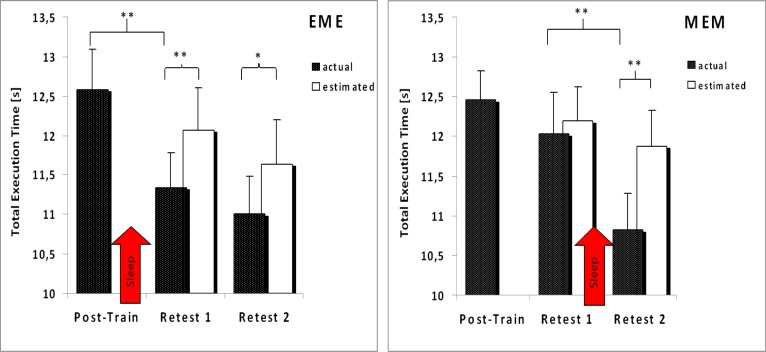
Total execution time (TET) at Post-Training (including block 8, 9 & 10), at Retest 1 (including block 11, 12 & 13) and at Retest 2 (including block 14, 15 & 16). Filled bars: actual data; open bars: estimated data. Presented are group means per test. Error bars: standard error of the mean. Left panel: EME-group; right panel: MEM-group.

Results are as follows: throughout the total 24-hrs retention period, TET significantly decreases in the EME-group (*F*_[Test]_ (1.315, 14.468) = 19.904, *p* < .001, η^2^_p_ = .644). As pairwise comparisons indicate, this is essentially due to considerable off-line improvements following the *first* 12-hr retention interval, i.e. after this group’s *sleep* period (Post-Training, Retest 1: *F* (1, 11) = 35.112, *p* < .001, η^2^_p_ = .761), while during the subsequent wake interval TET in this group does not indicate any significant alteration (Retest 1, Retest 2: *p* = .150, η^2^_p_ = .179). In the MEM-group, TET also significantly decreases throughout the total 24-hrs retention period (*F*_[Test]_ (2, 22) = 32.910, *p* < .001, η^2^_p_ = .749). And again, according to the pairwise comparisons, TET does not change significantly throughout this group’s wake interval (Post-Training, Retest 1: *p* = .127, η^2^_p_ = .199), but significantly decreases throughout its *sleep* period, which is the MEM-group’s second retention interval (Retest 1, Retest 2: *F* (1, 11) = 60.337, *p* < .001, η^2^_p_ = .846) (Figure 2 and 3).

### Comparing actual and estimated Retest-measures (execution time)

As was shown above, TET shows considerable and continuous improvement throughout acquisition in either group. Consequently, and different from the error rate, it has to be considered that TET may not have fully reached asymptotic performance at the end of initial training. In fact, in the EME-group TET decreases until the very last block of initial practice (Figure 2, left panel), while in the MEM-group, TET leveling off from block 9 to 10 might just reflect one of several transient performance fluctuations typical for this group (Figure 2, right panel: blocks 2 & 3, blocks 4 & 5). Therefore, continued practice, uninterrupted by retention intervals, possibly might have caused further improvements in performance quite similar to those actually reported at recall following sleep. Thus, it had to be determined, whether the significant reduction in TET (i.e. improvement in execution speed) for individuals of both experimental groups was indeed a result of sleep-related EC, or just a consequence of further practice.

One way to assess EC considering continued learning at retention, involves extrapolation of each subject’s respective initial training data. These *estimated* retention data are then used in conjunction with the individuals’ *actual* performance on the Retest trials. If the actual performance is better (i.e. TET lower) than the predicted performance, offline facilitation is assumed to have occurred. In the present study, estimated TET-Retest-measures were provided as follows: based on each single subject’s TET-acquisition data (means per trial block), for each individual a power function of the type y = *k*n^−c^ was calculated and used to obtain an estimate for that individual’s performance for the additional six trial blocks during both Retest 1 and Retest 2. Power functions are widely used to mathematically model practice-dependent changes in performance in the course of skill acquisition (Newell and Rosenbloom, 1981; Newell et al., 2009). Estimated TET-data for each individual then were collapsed across blocks in each test, thus providing mean predicted TET-performance at Retest 1 and Retest 2 for each one subject.

Thus, if sleep (but not wake) had indeed enhanced memory consolidation, *actual* TET should turn out significantly *lower* as compared to estimated TET in either group when tested right after the respective retention interval filled by sleep (Retest 1, EME-group; Retest 2, MEM-group). Also, we expected sleep-related performance facilitation to be preserved throughout an additional wake-period, which can be tested for at Retest 2 in the EME-group. According to these á-priori hypotheses, for each group one-tailed paired *t*-tests were calculated in order to compare actual and estimated TET-data at Retests.

As can be inferred from Figure 3, in each group the observed *actual* TET proves to be *significantly shorter* than the predicted TET *only after sleep* (EME_[Retest 1]_: *t* (11) = −3.901, *p*_[one-tailed]_ = .001, *d* = 1.13; MEM_[Retest 2]_: *t* (11) = −5.019, *p*_[one-tailed]_ < .001, *d* = 1.41). Also, in the EME-group this sleep-induced advantage of actual over estimated TET-performance appears to be preserved during the wake interval following sleep (EME_[Retest 2]_: *t* (11) = −1.967, *p*_[one-tailed]_ = .038; *d* = .57). In the MEM-group, however, actual and estimated TET-performance are not dissociated at all by the wake interval *preceding* sleep (MEM_[Retest 1]_: *t* (11) = −.825, *p*_[two-tailed]_ = .427, *d* = .25). According to these results, the significant reductions in TET reported for both experimental groups in the course of a 24-hr retention period cannot be attributed to merely continuing practice. Rather, and in support of our central hypothesis, they provide evidence for true sleep-related *offline-learning*, independently of sleep being administered during the first or during the second half of a 24-hrs retention interval.

## Discussion

As outlined in the introduction, previous findings on sleep-related EC in motor skill learning appear to be restricted almost exclusively to a specific set of fine motor tasks, namely sequential-finger-tapping skills (Albouy et al., 2013; Cohen et al., 2005; Doyon et al., 2009; Friedman and Korman, 2012; Fischer et al., 2005; Kuriyama et al., 2004; Lewis et al., 2011; Robertson et al., 2004; Walker et al., 2002; 2005; Witt et al., 2010). Earlier attempts at demonstrating sleep-related offline learning for motor tasks different from finger-tapping, for the most part came to nothing (Blischke et al., 2008). It was concluded, however, that at least certain *gross* motor tasks might be amenable to sleep-related EC, as long as they were sequentially structured, spatially defined, and explicitly acquired.

To test this notion, in the present experiment subjects practiced a complex sequence of 15 arm-movements with high spatial precision requirements. This task was executed on an electronic pegboard and involved a single end-effector (i.e. the non-dominant hand holding a small peg in a pincher-grip), while all three joints of the arm (i.e. wrist, elbow, and shoulder) were to move freely. End-point locations of the sequence’s elemental reaching-movements formed a two-dimensional spatial pattern. Subjects were to avoid any sequence errors (ES), and at the same time to minimize total execution time (TET) per trial. Visual guidance was provided during sequence execution on a computer screen, but there was no additional feedback on performance measures. Initial training of this task was administered either in the evening (EME-group) or in the morning (MEM-group). Subsequently, subjects in either group underwent two retests 12 (Retest 1) and then again 24 hrs (Retest 2) later. During retention, individuals in each group slept at night, and kept awake during the respective daytime interval. It was expected that sleep, but not wake, would enhance performance at retention beyond any improvement merely being afforded by exposure to additional practice trials.

As it turned out, *performance error (ES)* was rather low for individuals for both groups right from the start, and did not improve any more throughout initial training. Mean ES then significantly decreased during the sleep-filled retention interval (i.e. from Post-Training to Retest 1) in the EME-group only, but remained unchanged throughout the total 24-hr retention period in the MEM-group. So there is only partial evidence for sleep-related EC on account of the error data, but this latter finding may not be surprising given the small number of errors over all. Obviously, subjects closely followed instructions to keep the error rate at bay, and not to increase execution speed at the cost of increasing number of errors at the same time. In fact have error measures (as opposed to the respective speed measures) repeatedly been proven to be less or even non-sensitive to sleep-related EC also in sequential-finger-tapping tasks (Albouy et al., 2013; Doyon et al., 2009). In any case the present results clearly indicate that the between-session changes in performance speed referred to below did not occur at the expense of performance accuracy.

In contrast *Total Execution Time (TET;* non-erroneous sequences only), an index of *performance speed*, showed considerable improvement for all individuals in each group throughout initial training without ever leveling off (no group differences), thus rendering continued learning even at retests highly possible. During retention, mean *actual TET* in each group *significantly decreased* again, when being retested *following sleep* (i.e. at Retest 1 in the EME-group; at Retest 2 in the MEM-group), but always remained stable throughout the respective wake interval. Although this result appeared to be well in line with the notion of sleep-related EC, in order to arrive at a definite conclusion here continued learning at retests first had to be ruled out as an alternative interpretation of these data. To this end, TET measures to be expected at retests if practice had continued uninterrupted by retention intervals were estimated by means of power functions derived from each individual’s initial training data. These predicted TET-data then were compared to the actual TET-data established at retests, which procedure yielded the following results: when retested *following sleep*, in both groups *actual* TET turned out *significantly shorter* than *predicted* TET, while both measures did not differ statistically just following wake in the MEM-group. This result confirms that actual TET-reductions found at retests following sleep, cannot be attributed merely to continued learning, but rather reflect some EC.

Given the fact that visual guidance was provided during sequence execution in our experiment, the present results might reflect not only effects of sequence memory enhancement, but also of sleep-related improvements of mechanisms relevant to the online processing of visual stimulus information. Our results could be more purely related to EC of the sequence representation if directly after the practice sessions as well as during retests following the respective retention intervals, subjects had also produced the arm movement sequence without any visual instruction stimuli in a free recall condition, and yielded the same results. We did not introduce such a testing procedure in our present study in order to avoid possible confounds if the same subjects underwent different retest conditions (i.e. with and without visual guidance). However, we have recently applied this procedure to a follow-up study, which is still in progress. From this study, twelve subjects (all young adults) so far have been analyzed. They initially practiced a ten-element arm movement sequence on the peg-board in the morning under visual guidance (120 trials), and then reproduced the same sequence under free recall conditions 15 minutes (Post-Training), 12 hrs (Retest 1) and – after a night of sleep - 24 hrs later (Retest 2). Eight participants were able to freely recall the sequence even 24 hrs after acquisition. Preliminary results on these eight subjects show about the same pattern of sleep-related EC with respect to TET as reported in the present paper (Post-Training, Retest 1: *p* = .922, η^2^_p_ = .001; Retest 1, Retest 2: *F* (1, 7) = 8.269, *p* = .024, η^2^_p_ = .542). Also, performance in the free recall early retention test (i.e. Post-Training) was not different from that during the last three blocks of practice with visual sequence information provided (*p* = .919, η^2^p = .002). We therefore conjecture the findings of EC presented in the present paper also to express over-night enhancement of s*equence memory* rather than facilitation of online processing of visual stimulus information.

It has been questioned previously whether performance improvements following sleep are real or whether such changes rather reflect time-of-day confounds (e.g. compensation of fatigue effects) due to the experimental protocols (Brawn et al., 2010; Cai and Rickard, 2009). However, in the present study the extent of learning during acquisition and performance levels at the end of training were quite similar in both groups, regardless of whether the training took place in the evening or in the morning. This then suggests that the expression of delayed sleep-related post-training gains reported here are indeed due to the EC process rather than the mere result of fatigue compensation taking place over night. This does not completely rule out circadian confound, however, as one might argue the EC process to be related rather to circadian influences than to the experience of sleep as such. All the same, empirical evidence to date does not support any such notion: first of all, there is abundant evidence for EC being correlated either with certain sleep parameters or (in general to a lesser extent) with the elapse of time (Fischer et al., 2005; Walker, 2005), while there is no indication whatsoever of certain circadian (i.e. time-of-day) aspects being of relevance here. Secondly, sleep-related EC has repeatedly been shown for the serial-finger-tapping task following day-time sleep, thereby incorporating experimental paradigms better suited for controlling for the time-of-day difference between acquisition and test sessions (Albouy et al., 2013; Doyon et al., 2009). And, last but not least, we found an increase in performance in both experimental groups only after sleep but not after the wake period, thus ruling out that EC was significantly influenced by retention interval duration.

One might also ask if the above findings on sleep-related EC might be biased by our participants’ dexterity, since they carried out the present experiment with their non-dominant arm. In this respect in the present study we merely followed the standard procedure applied by the vast majority of studies on sleep-related EC. Surprisingly, in the literature surveyed we did hardly find any comment on this procedure, let alone an explicit justification. Yet, there is indeed a theoretical possibility that sleep may interact differentially with the hand or arm used during motor sequence learning. To date, however, there is hardly any evidence to support such an assumption. To our knowledge only one study so far involved two groups of subjects who practiced a novel finger opposition sequence either with their dominant or with their non-dominant hand, and were retested 24 hrs later (Balas et al., 2007). In general, results of that study suggested that training of either hand may trigger delayed gains in performance. Baseline data presented by Balas et al. (2007) for each of the two groups enrolled in their Experiment 1, however, do not rule out the possibility of slight advantages of the dominant over the non-dominant hand in this respect. Unfortunately this aspect was not subjected to any statistical proof in that study.

All in all, we believe our results to be well in accord with the above hypothesis, namely that even in a *gross* motor task involving a sequence of coordinated limb movements, sleep following initial learning significantly facilitates performance. This finding at the same time successfully extends the notion of sleep-related EC beyond the standard finger-tapping paradigm. It has to be stated, however, that the present finding only was achieved after deliberately increasing task difficulty (in terms of precision requirements) as well as task complexity (in terms of sequence length), as compared to an earlier attempt in scrutinizing sleep-related EC in an arm-movement sequence (i.e. Schmidt et al., 2010). However, at this point it cannot yet be determined to what extent either of these features (i.e. task complexity reflecting memory load, or spatial precision requirements reflecting motor control demands) specifically contributed to the results presented here.

So undoubtedly, more and detailed information is needed on what task features precisely are essential for sleep-related EC to come into effect in gross motor skills. Only then could this concept truly be of relevance in the various applied fields of motor sciences. Some further leads that might help identifying task features relevant in this context could perhaps be derived from a very recent study by Kempler and Richmond (2012), which came to our knowledge only after the experiment presented here was completed already. Encouragingly these authors, too, found sleep-related offline learning in a gross motor task. Their task required participants to complete a sequence of six different body configurations, with each configuration defined by the two arms pointing simultaneously into the same or different directions (i.e. straight up or down or 90° sideways) in the frontal plane. So actually this task in a way mimicked a signalman transmitting a message through a series of flag signals. This sequence had to be repeated continuously over periods of 30 seconds during training and retests. Dependent measure was the average number of accurate (half)cycles (i.e. three correct configurations in a row) per each 30 s trial block as an index of speed.

Kempler and Richmond in their study did not report any quantitative criteria for correct angular positioning (i.e. up, down, or sideways) of their subjects’ arms. Thus spatial precision requirements presumably were rather low, as was sequence length, which may have considerably reduced control demands as well as memory load of their task. And yet these authors found sleep-related offline learning all the same. This at first sight seems to be at odds with our above supposition, according to which EC can be reliably shown in gross motor tasks only if task difficulty is set at a sufficiently high level. However, while Kempler and Richmond followed the same experimental protocol as we did in our present experiment, sample sizes involved in their study were about three times those of ours (i.e. 35 as compared to 12 participants per experimental group). With sample sizes that large, even a seemingly small effect (due to only moderate task difficulty) might have turned out as statistically significant. Unfortunately, Kempler and Richmond did not report any effect sizes.

On the other hand, the Kempler and Richmond task required participants to *simultaneously* produce heterogeneous movements with both arms at transitions between the elementary sequence positions. This task feature perhaps induces a dual-task requirement as long as each arm’s position has to be specified separately, a process that virtually promotes positioning errors or at least prolongs execution time, because at an early learning stage the positioning responses for both arms cannot yet be selected and prepared at the same time. This interpretation clearly draws on the concept of a “response selection/preparation”-bottleneck, a concept well established by researchers utilizing the *P*sychological *R*efractory *P*eriod (PRP) paradigm, which was originally introduced by Harold Pashler (Pashler and Johnston, 1998). At later learning stages and sufficient practice, however, the respective motor actions to be taken by either arm at each transition between sequence elements should eventually be integrated (“chunked”) into one-and-the-same memory representation. Progressive chunk formation of this kind ought to reduce interference at recall, and help speeding up sequence execution (Rhodes et al., 2004; Verwey, 1996). To some extent, such internal chunking might also have taken place offline during the sleep-filled retention intervals, resulting in the respective sleep-related improvements of performance observed by Kempler and Richmond (2012).

Indeed, some evidence of sleep-related chunking has been provided by Kuryiama et al. (2004) already as far as the sequential-finger-tapping task is concerned. This was achieved by demonstrating significant overnight improvement of subjects’ slowest key-press transitions, which was not observed during acquisition, nor following a day-time wake interval. Interestingly, these authors also found the greatest amount of sleep-related offline improvement (i.e. increased execution speed) for just that task configuration, which incorporated the highest level of task complexity (i.e. sequence length) and the greatest degree of between-limb coordination (i.e. bimanual task execution involving all eight digits). For an explanation, the authors argue that the cerebral network size of the sequence’s memory representation, and thereby the potential for sleep-dependent synaptic plasticity, would increase considerably with the number of digits (or in general end-effectors) involved in cooperative movement production.

As matters stand it cannot be decided whether Kempler and Richmond’s findings are owing to their large sample sizes or rather to offline integration of elementary memory components and/or improvements in between-limb coordination. So with respect to gross motor tasks, the relation of certain task characteristics (like e.g. task difficulty, task complexity, and demands on between-limb coordination) and sleep-related EC is still up to discussion, as is the question to which extent chunking processes actually contribute to offline improvements in skilled motor performance (Kuriyama et al., 2004; Wright et al., 2010). While these questions are presently addressed by our group, certainly more research will be needed to this end.

Another point at issue relates to the *type* of motor sequence representation, which is supposed to be enhanced offline while subjects are asleep. According to a widely accepted model proposed by Hikosaka and his colleagues (1999), the acquisition of sequential behaviors resides in the interaction between different neural networks that would encode the same motor sequence in two different coordinate systems (i.e., spatial and motor). One memory component is thought to incorporate allocentric (spatial) coordinates, and to constitute an abstract effector-independent representation of a series of movements that need to be executed in an external frame of reference. The other memory component is supposed to be mediated through egocentric (motor) coordinates, and thus to constitute an effector-dependent, movement-based skill realized in an internal frame of reference (Hikosaka et al., 1999).

Hikosaka’s model has also been applied to research on motor memory consolidation. For explicitly learned finger-tapping sequences it has been shown conclusively, that *sleep* specifically favors enhancement of the *extrinsic* (spatial) sequence representation, while consolidation of the respective *intrinsic* (motor) representation was *not* modulated by the sleep/wake condition (Albouy et al., 2013; Witt et al., 2010). While Hikosaka’s integrative model proposes that the spatial component is created rapidly early during training with the motor component developing more slowly with extended practice, according to Albouy and colleagues’ results both spatial and motor representations exist already after minimal training, with only the former being amenable to sleep-related EC. However, the question arises as to what extent this dissociation also applies to gross motor tasks, where motor control requirements typically exceed those encountered in sequential-finger-tapping skills.

Furthermore, the laterality issue addressed above still remains of interest in the light of recent findings on the inter-limb transfer of multiple-element sequences. Here, non-dominant to dominant limb transfer has been shown to be superior to dominant to non-dominant limb transfer, if an additional load was added during acquisition, and if transfer tests required end-effector movement to the same spatial positions that had been practiced during acquisition (i.e. no mirror movements) (Panzer et al., 2010). For unloaded sequences, however, this type of effector transfer has been proven to be symmetrical (Kovacs et al., 2009). Asymmetric inter-limb effector transfer thus appears to be tied up with specific task requirements, here those concerning the control of movement dynamics. These and other findings (Sainburg, 2005) give rise to theoretical models of hemispheric specialization. In the end and with respect to specific task requirements, such models might also be of some relevance to concepts of motor memory consolidation.

It should be noted that polysomnographic data acquisition and analysis were not within the scope of the present study. Therefore, the present data do not allow for any conclusions regarding a particular sleep stage promoting EC of gross motor skill representation. With respect to the allocentric sequence representation of sequential-finger-tapping skills, there is increasing evidence for sleep-dependent gains in performance being correlated with the density of NREM sleep spindles and NREM stage 2 sleep duration (Albouy et al., 2013; Barakat et al., 2011; Walker et al., 2002; Witt et al., 2010). Other authors, however, have found this type of sequence representation (and declarative memory consolidation in general) to be correlated with REM sleep duration (Cohen et al., 2005; Fogel et al., 2007). Considering then gross motor tasks again, this question also calls for closer inspection in future research.

In conclusion, the present findings again successfully extend the notion of sleep-related EC to a gross motor task, i.e. a complex arm movement sequence. At the same time we are confident that our experimental paradigm is also well suited to address systematically and in some more detail some of those questions discussed here. While suchlike intents would be primarily related to the field of basic research, our findings also bear some practical implications relevant to sport and rehabilitation, whenever gross motor tasks have to be learned or relearned efficiently. This might specifically pertain to complex skills involving the upper extremities and requiring fast and precise execution of a series of sub-movements in Euclidian space. Here, we would recommend practice to be distributed over two or more sessions separated by night or daytime sleep, respectively. Distributed practice schedules of this kind might be especially beneficial in situations, where the amount of practice per session is limited of necessity, as is often the case in rehab-training or acquisition of sport skills requiring extraordinary high physical or mental effort.

## Figures and Tables

**Picture 1.**
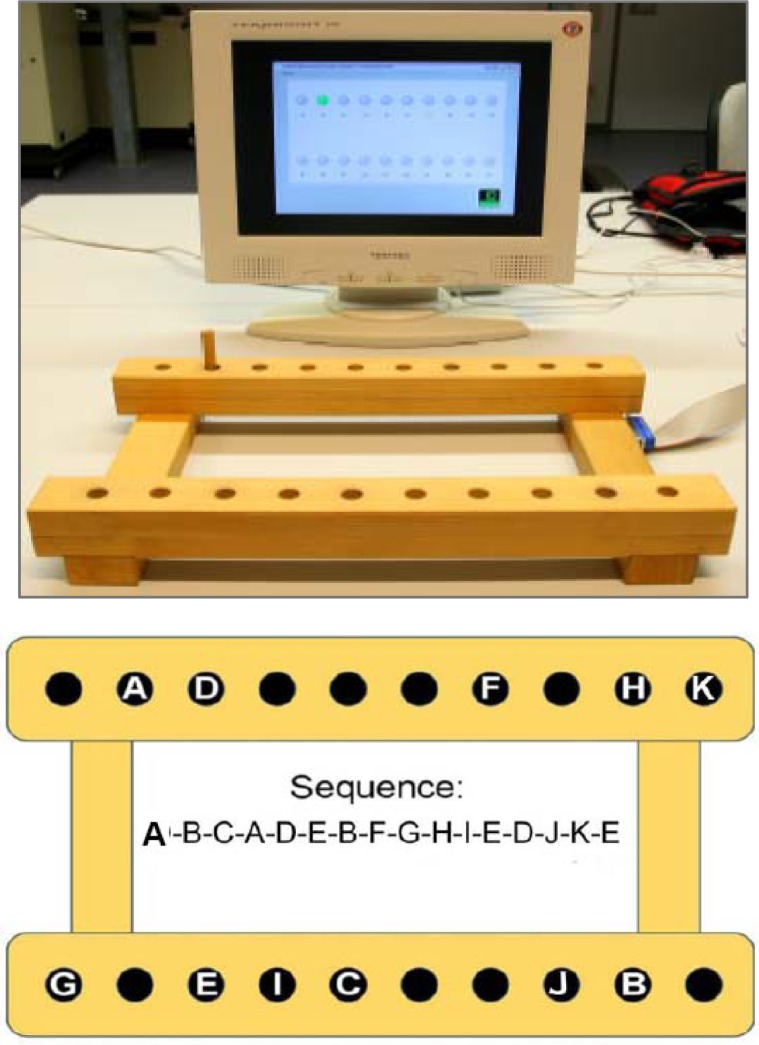
Experimental apparatus (upper panel), and spatial locations to be reached for one after the other, defining the fifteen-element arm movement sequence (lower panel).
